# Effectiveness and cost-effectiveness of telephone-based cognitive-behavioural therapy in primary care: study protocol of TIDe – telephone intervention for depression

**DOI:** 10.1186/s12888-017-1429-5

**Published:** 2017-07-19

**Authors:** Birgit Watzke, Elisa Haller, Maya Steinmann, Daniela Heddaeus, Martin Härter, Hans-Helmut König, Karl Wegscheider, Thomas Rosemann

**Affiliations:** 1 Department of Psychology – Clinical Psychology and Psychotherapy Research, University of Zurich, Binzmühlestrasse 14/16, Zurich, Switzerland; 2 Department of Medical Psychology, University Medical Center Hamburg-Eppendorf, Martinistraße 52, Hamburg, Germany; 3 Department of Health Economics and Health Services Research, University Medical Center Hamburg-Eppendorf, Martinistraße 52, Hamburg, Germany; 4 Institute for Medical Biometry and Epidemiology, University Medical Center Hamburg-Eppendorf, Martinistraße 52, Hamburg, Germany; 5 Institute of Primary Care, University of Zurich, Pestalozzistrasse 24, Zurich, Switzerland

**Keywords:** Telephone-delivered psychotherapy, Evidence-based treatment, Short-term CBT, Primary care, Access to mental health services, Mild to moderate depression

## Abstract

**Background:**

Despite the availability of evidence-based treatments for depression, a large proportion of patients remains untreated or adequate treatment is initiated with delay. This situation is particularly critical in primary care, where not only most individuals first seek help for their mental health problems, but also depressive disorders – particularly mild to moderate levels of severity – are highly prevalent given the high comorbidity of chronic somatic conditions and depression. Improving the access for evidence-based treatment, especially in primary care, is hence a priority challenge in the mental health care agenda. Telephone usage is widespread and has the potential of overcoming many barriers that individuals suffering from mental health problems are facing: Its implementation for treatment delivery presents an option for optimisation of treatment pathways and outcomes.

**Methods/design:**

This paper details the study protocol for a randomised controlled trial (RCT) evaluating the effectiveness of a telephone-administered short-term cognitive-behavioural therapy (T-CBT) for depression as compared to treatment as usual (TAU) in the Swiss primary care setting. The study aims at randomising a total of 216 mildly to moderately depressed patients, which are either identified by their General Practitioners (GPs) or who self-refer to the study programme in consultation with their GP. The trial will examine whether telephone-delivered, manualised treatment leads to clinically significant reduction in depression at follow-up. It will further investigate the cost-effectiveness and acceptability of the intervention in the primary care setting.

**Discussion:**

Conducting a low-intensity treatment on the telephone allows for greater flexibility for both patient and therapist, can grant more anonymity and can thus lead to less hesitation in the patient about whether to attempt treatment or not. In order to benefit from this approach, large-scale studies need to prove superior effectiveness and cost-effectiveness of telephone-delivered therapy over routine care for patients with mild to moderate depression.

**Trial registration:**

ClinicalTrials.gov NCT02667366. Registered on 3 December 2015.

## Background

Depressive disorders are a common mental health condition with a high prevalence and burden of disease. With a 12-month prevalence of approximately 8% it is one of the most widespread mental disorders [1] and causes a high degree of personal suffering and impairment. It is also associated with high societal costs, thus presenting a substantial challenge to the health care system, not least in view of the considerable direct and indirect costs implied [2]. In Switzerland, for example, the total cost caused by depression on a national level amounts to more than 8 billion Swiss Francs per year [3]. Estimations predict a further increase in depression-related disease burden in the next 20 years; depression will represent the second most important factor for impairment and premature mortality after cardiovascular diseases in highly economically developed countries [4].

**Mild to moderate depression** presents a particular challenge to the health care system for various reasons. Firstly, there is a high prevalence of these levels of depression among the general population. In a national representative data sample in the US, an overall prevalence of 20% of any depressive symptoms was assessed, the majority of which were mild to moderate depressive symptoms [5]. Secondly, the nature of mild to moderate depressive episodes involves the risk of exacerbating symptoms and developing chronic or more severe forms of the depressive disorder if they remain untreated, are inadequately treated or if treatment is initiated with delay [6]. In order to treat depressive disorders efficiently and in order to avoid deterioration of the individuals’ condition and increase of the societal burden, the provision of timely and adequate treatment is of great importance. Therefore, inappropriate health care for patients suffering from depression poses a third challenge for the health care system: according to international trials, treatment adequacy is particularly low in mild to moderate degrees of depression [7]. In primary care, almost two thirds of patients suffering from mild depression and one third of patients suffering from moderate depression receive exclusively antidepressant medication [6], even though international guidelines recommend psychotherapy and low-intensity treatment – with a particular focus on cognitive-behavioural therapy (CBT) – as first-line treatment [8, 9]. These clinical guidelines further explicitly advise against the application of antidepressant medication in mild degrees of severity except for special situations, due to the unfavourable risk-benefit ratio with regard to undesirable side effects. Treatments that are not compliant to clinical guidelines consequently present a serious problem for patients suffering from less severe depressive disorders.

However, initiating adequate psychological treatment is impeded by various barriers to access care on an individual level (fear of stigma, lack of time, comorbid somatic conditions, cultural factors), on the provider level (inadequate diagnostics and knowledge about mental disorders in primary care, lack of time) and on the systemic level (waiting lists, insufficient interconnectedness between health care professionals, insufficient treatment capacity) [10].This inadequate supply situation particularly affects primary care, given the high prevalence rates of depression there (ranging between 5 and 30%) [11] with general practitioners (GP) being in charge of detecting patients, motivating them for treatment and initiating it. The latter comprises either treatment in primary care or the referral to specialized care, which is rather seldom (e.g. in Switzerland only approx. 13%, (e.g. [12])).

Given these challenging tasks in (primary) care, low-threshold and intensity-appropriate (so-called `low intensity´) formats of psychological treatments, such as telephone-administered psychotherapy, may contribute to a better access to evidence-based treatment.

### Telephone-administered psychotherapy

One increasingly promising approach to overcoming the aforementioned barriers is the application of treatments with the help of remote communication. Telephone and particularly mobile phone is an omnipresent and well-accepted means of communication [13], which has become a reliable tool for managing and delivering care in many areas of the health care system and for a variety of somatic conditions [14, 15]. Telephone-based interventions focusing on mental health have further been proven beneficial and effective in providing CBT for a variety of mental disorders and symptoms, including anxiety disorders in older patients [16], insomnia, health-related quality of life and disability [17] and a variety of conditions where mental health problems co-occurred with chronic somatic disorders. Telephone-delivered psychotherapy – with a special emphasis on CBT (T-CBT) – also shows promising results for the treatment of depression in international meta-analyses [18]. Trials that have thus far investigated this approach for depression in primary care settings have shown lower attrition rates and equivalent improvement in symptom severity at posttreatment compared to face-to-face treatment [19, 20]. Thus, T-CBT could lead to expanded, more flexible, and accessible psychotherapeutic services with the additional option of lowering costs.

However, the majority of trials aim at the reduction of depressive symptoms in patients suffering from chronic and distressing somatic conditions, such as HIV/AIDS, multiple sclerosis and cancer [21–23]. Other studies do not examine T-CBT as a stand-alone treatment for depression; for example, some studies focus on T-CBT in depression care management [24, 25] or as adjunct support in order to ensure adherence to pharmacological treatment. It is also insufficiently clarified whether T-CBT has long-lasting effects on the patients’ mental health and if long-lasting effects would pertain to a less severely depressed patient sample [19].

Despite promising results for T-CBT for depression in primary and specialist care [18, 26], it is necessary to investigate an intensity-appropriate therapy delivered via telephone as a stand-alone intervention as well as to examine effects that go beyond the post-treatment improvement for patients with mild to moderate depression. Lastly, large-scale effectiveness studies with more specific patient samples are required to add to a solid evidence-base and need to be adopted to the specific characteristics of various health care systems. The proposed trial enables us to investigate whether T-CBT as a stand-alone treatment element can improve the acute treatment of mild to moderate depression, which will be undertaken in the Swiss health care system.

### Objectives

Our study aims at evaluating the effectiveness of a telephone-delivered CBT as an add-on treatment to routine care in primary care patients with mild to moderate depression under clinically representative conditions. Secondary objectives include the evaluation of T-CBT’s cost-effectiveness compared to treatment as usual (TAU). Further objectives are the examination of process variables. Particularly, patients’ and therapists’ acceptance and satisfaction with the telephone-intervention as well as process quality of the treatment and therapeutic alliance will be investigated.

### Hypotheses

Primary hypothesis is that the addition of T-CBT to routine care is superior to routine care alone (treatment as usual, TAU) in reducing depression symptom severity measured with the Patient Health Questionnaire (PHQ-9) at 12-month follow-up. Main secondary hypotheses are that T-CBT is more effective at reducing symptom severity at end of treatment (t1) and that T-CBT is cost-effective compared to TAU.

### Ethical approval

Approval for the study has been obtained from the responsible local Ethics Committee of the Canton of Zurich (Ref. Nr. 2015–0417). The study will be conducted according to the principles of the Declaration of Helsinki and in line with the Guidelines for Good Clinical Practice (GCP).

## Methods: Participants, interventions, and outcomes

### Study design

The proposed study is a prospective randomised controlled effectiveness trial of short-term T-CBT compared to treatment as usual in primary care patients with mild to moderate depression. Both study arms will receive treatment as usual (TAU). A total of 216 individuals who fulfil inclusion criteria are intended to be randomised in either active treatment or control condition with a 1:1 allocation. Outcomes will be assessed at baseline (t0), at the end of treatment (t1, i.e. 4 months after baseline in CG) and 12 months (t2) after baseline. Table 1 presents a structured summary of the trial by providing items from the WHO Trial Registration Data set.

**Table 1:** WHO Trial Registration Data Set

Data category	Information
Primary registry and trial identifying number	ClinicalTrials.gov NCT02667366
Date of registration in primary register	3 December 2015
Sources of monetary or material support	Gottfried and Julia Bangerter-Rhyner-Foundation (Bangerter-Stiftung) and Swiss Academy of Medical Sciences (Schweizerische Akademie der Medizinischen Wissenschaften, SAMW)
Primary sponsor(s)	Prof. Birgit Watzke, University of Zurich
Contact for public queries	M.Sc. Elisa Haller Department of Clinical Psychology and Psychotherapy Research Binzmühlestrasse 14/16, CH-8050 Zürich Switzerland e.haller@psychologie.uzh.ch
Contact for scientific queries	Prof. Dr. Birgit Watzke Department of Clinical Psychology and Psychotherapy Research Binzmühlestrasse 14/16, CH-8050 Zürich Switzerland b.watzke@psychologie.uzh.ch
Public title	Telephone-Intervention/Information for Depression (TIDe)
Scientific title	Effectiveness and cost-effectiveness of a telephone-based cognitive-behavioural therapy in primary care for mild to moderate depression
Countries of recruitment	Switzerland
Health condition(s) or problem(s) studied	Mild to moderate depression
Intervention(s)	Telephone-delivered short-term cognitive-behavioural therapy
Key inclusion and exclusion criteria	Inclusion criteria: Age ≥ 18 years, mild to moderate depression according to ICD-10, PHQ-9 > 5 and ≤15, able to fill in questionnaires, German language Exclusion criteria: suicidality or suicidal tendency, currently or in last 3 months in psychotherapeutic treatment, chronic depression or dysthymia, severe depression, psychopharmacological medication that is not stable for 3 months prior to enrolment.
Study type	Randomised-controlled superiority trial
Date of first enrolment	January 2016
Target sample size	216
Recruitment status	Recruiting
Primary outcome(s)	Symptom severity at t2 (PHQ-9)
Key secondary outcomes	Symptom severity at t1 (PHQ-9), Response defined as 50% reduction from t0 to t1,remission defined as <5 points in the PHQ-9 at t2 and t1, relapse defined as ≥5 points in the PHQ at t2 following remission at t1, cost-effectiveness

### Participants

The trial aims at randomising a total of 216 patients with mild to moderate depression, who are either recruited by a participating GP in Zurich or who take notice of the study advertisement in the media (esp. regional newspapers) and thus self-initiate study participation in consultation with their GP.

#### Inclusion criteria

Eligible study participants need to meet the following inclusion criteria: a) age of at least 18 years, b) PHQ-9 score of >5 and ≤15, c) a diagnosis of mild or moderate depression according to ICD-10, more specifically a single episode major depressive disorder or recurrent depressive disorder with a mild or moderate degree of severity (i.e., F32.0, F32.1, F33.0, F33.1) and d) given informed consent.

#### Exclusion criteria

The following criteria lead to exclusion from the study: a) chronic depression i.e. symptoms of at least 2 years duration, b) suicidality, suicidal tendency or suicidal ideation, c) insufficient knowledge of the German language, d) taking part in psychotherapeutic / psychological treatment currently or in the last 3 months, e) a physical or mental condition that does not allow the completion of questionnaires. Psychopharmacological treatment is only an exclusion criteria, if medication is not stable for at least 3 months prior to study inclusion.

### Study interventions

#### T-CBT (intervention group)

The intervention group (IG) will receive T-CBT in addition to TAU, which is a short-term telephone-delivered psychotherapy. The therapy programme is a manualised cognitive-behavioural therapy (CBT) consisting of one initial face-to-face appointment and 8–10 subsequent telephone sessions (+ 2 optional booster sessions) with the study therapist. Telephone contacts will take place on a weekly and – after the first four sessions – on a bi-weekly basis and will last 3–4 months in total. Each telephone contact should last between 30 and 40 min and will be audio recorded for the sake of assessing treatment integrity and evaluating treatment process variables.

The therapy programme is a translated and culturally adapted version of the programme *Creating a balance* developed in the US [24, 27]. The German version (“Ins Gleichgewicht finden”) has been evaluated as part of a stepped care trial [28], i.e. was part of a complex intervention of stepped care which was evaluated as a whole. For the proposed study, the manual has been adapted slightly according to special characteristics and cultural features of the Swiss population (especially adaption of the case studies, e.g. using typical Swiss expressions and names).

The manualised programme comprises the central components of CBT for depression: the first part of the programme focuses on behavioural activation and the subsequent chapters centre upon strategies of cognitive restructuring. Further essential treatment elements are psycho-education and relapse prevention. After a maximum of 10 sessions there is the option of adding up to two booster sessions for enhancing long-term maintenance of treatment effects. Patients receive a workbook which they work with independently between sessions and then discuss materials with the telephone therapist. Moreover, since regular symptom monitoring (PHQ-9) is part of the telephone treatment, patients learn to monitor themselves concerning their depressive state. Despite the strong structure and guidance provided by the therapist, the programme has a strong self-help character and thus requires patients to delve into the contents and acquire skills themselves with support of the therapist.

T-CBT will be conducted by three study therapists in training, who are clinical psychologists and are in CBT training with a minimum of 2 years of experience in the treatment of patients predominantly suffering from affective disorders. The study therapists receive training in T-CBT and supervision will be provided every month by a senior study team member (BW).

Adverse events will be regularly and systematically monitored by the telephone therapist. Emergency plans will be prearranged in case of acute suicidality, e.g. calling the emergency services. In case of an emotional decompensation there will be the possibility to meet in a face-to-face session or to choose a more intensive treatment (e.g. inpatient treatment).

#### Text messages (control group)

Patients in the control group (CG) will receive TAU and a minimal intervention additionally: This minimal intervention entails a total of 10 text messages which contain factual information on depression. The text messages will be sent to the patient on a weekly and later bi-weekly basis, i.e. in the same frequency as telephone-sessions in the IG. The receiver cannot and is not required to reply to the messages. The messages contain short neutral information on different aspects of depressive disorders, for example on prevalence, clinical picture, course and the social implications of the depressive disorder (e.g. “Depression is very common. More than every fifth person experiences depression once in their life”). The text messages should be of interest for the patients in the control condition without having a therapeutic effect. The contents of the text messages differ from a psychoeducational intervention in the sense that they a) do not follow a systematic and structural knowledge transfer and b) do not convey strategies that enable a health-promoting lifestyle. The minimal intervention is amended to TAU to control for effects resulting from purely contacting patients and reminding them. It can be assumed that they might have a minimal effect on patients on the short term, but not a lasting (therapeutic) one.

### Outcomes and measures

#### Outcome assessment

Outcomes will be assessed via self-report paper-pencil questionnaires at baseline, post-intervention and follow-up, which will be sent to the patient via mail. The primary outcome of effectiveness refers to a change in symptom severity from t0 (baseline) to t2 (12 months after baseline), measured by the German version of the PHQ-9.

Secondary outcome parameters will include the change in depressive symptoms from t0 to t1, response defined as a 50% reduction in the PHQ-9 value from t0 to t1, remission defined as <5 points in the PHQ-9 at t2 and t1, and relapse defined as ≥5 points in the PHQ-9 at t2 following remission at t1. Further secondary outcome is change in health-related quality of life (HrQoL) assessed by the SF-12. Furthermore, the incremental cost-effectiveness of T-CBT compared to TAU will be analysed based on (direct and indirect) costs and quality-adjusted life years (QALYs) observed within the 12-month follow-up period.

In addition to outcome measures, there will be an assessment of structural and process variables. For the IG, these will entail amount and duration of telephone contacts as well as contents of the sessions. Additional assessments take place at the beginning of each telephone session in order to monitor symptom severity and measure therapist ratings regarding the therapeutic alliance between patient and therapist. Therapists’ and patients’ acceptance of and satisfaction with T-CBT will be assessed with a self-constructed questionnaire based on instruments from the study team’s prior research.

#### Outcome measurements

**Patient Health Questionnaire (PHQ-D):** is the German version of a patient rating instrument covering the severity and frequency of symptoms of depression (9 items), generalised anxiety syndrome (7 items), somatoform syndrome (13 items) and panic syndrome (11 items). The instrument demonstrates high levels of validity in the diagnosis and evaluation of symptom severity of major depressive disorder in primary care patients [29]. The PHQ-D is a well-accepted, reliable instrument and shows diagnostic validity [30]. The depression subscale (PHQ-9) covers severity/frequency of the primary and secondary symptoms of major depressive disorder [29] and has proven reliability and validity for the use in primary care [31].

**Short Form − 12 (SF-12):** is the short version of the Short Form 36 Health Survey and assesses overall health-related quality of life. The 12 items constitute the two subscales physical and mental health. It is an internationally well-established instrument with proven psychometric properties [32].

**General Self-Esteem Scale (GSES):** is a revised version of the Rosenberg Self-Esteem-Scale and captures global, unidimensional self-esteem. Ten statements regarding beliefs about personal ability, value and attitudes toward oneself are rated on a 4-point Likert scale. Reliability and validity show satisfactory values [33].

**General self-efficacy scale (GSE):** is a one-dimensional self-rating scale measuring the global optimistic beliefs about one’s self [34]. On a four-point Likert scale, subjects indicate their level of agreement with ten statements about the self-related expectation of coping with a difficult situation.

**Depression-related self-management behaviour:** this five-item scale was created to examine the respondents’ perceived ability to cope with depressive symptoms and complaints [35] and to assess to which extent patients integrate pleasant activities in their daily lives.

**Client Sociodemographic and Service Receipt Inventory (CSSRI):** will be employed to measure health care utilisation and productivity loss [36]. A slightly adapted version of the instrument for the Swiss health care system will assess the type and frequency of all health services the respondent used as well as absence from work during the last 6 months.

**EQ-5D-5 L:** Health-related quality of life for the economic analysis will be measured using the EQ-5D-5L, an internationally established instrument to derive utilities (EQ-5D index) for cost-effectiveness analyses [37] (www.euroqol.org).

**Psychotherapy Motivation Questionnaire (FPTM-23):** is the short version of the Questionnaire on Psychotherapy Motivation [38], a measure used to assess the motivation for undertaking a psychotherapy. Three out of six sub-scales – psychological stress, hope and denial of need for help – are used in this study. These sub-scales also indicate sufficient and good internal consistency [38].

### Participant timeline

**Fig. 1:**
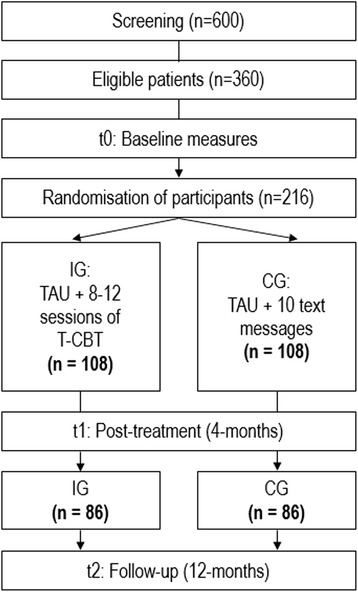
SPIRIT flowchart of study participants

The SPIRIT flow diagram of enrolment, pre-study screening, interventions and assessment is provided in Fig. 1.

### Sample size

With a total sample size of 172 evaluable patients (IG: *n* = 86; CG: *n* = 86), a medium effect size (d = .44) can be detected in the primary outcome measure between T-CBT and TAU in the planned ANCOVA with a two-sided hypothesis, significance level of 0.05 and statistical power of 90% (when error variance is reduced to 20% by the covariate (baseline depressive symptoms)). With 20% loss-to-follow-up, 216 patients need to be randomised for both ITT and complete-case analysis to reach a power of 90%. Since prior projects suggest that approx. 60% of the patients at risk will meet the inclusion criteria and that again roughly 60% of depressive patients in primary care will participate in the study, 600 patients will be screened for inclusion.

### Recruitment and study procedure

The recruitment process entails two main strategies. In the first instance, study patients will be recruited by 15 participating GPs in the area of Zurich, Switzerland. All patients visiting their GP who are at risk for depression, especially those who present diffuse somatic or chronic somatic symptoms [8] will be screened for depressive symptoms. The GP who has been trained by the study team has two alternative ways of proceeding with study inclusion: a) the GP carries out diagnostics and conducts study enrolment by checking eligibility criteria, obtaining the consent form and handing out the baseline questionnaire; b) the GP alternatively refers the patient directly to the study team: If the patient consents to being contacted, a member of the study team will call the patient and will carry out ICD-10 diagnosis and study enrolment over the telephone. Baseline questionnaire and consent form will be sent via mail.

The second strategy of participant recruitment will be patient-initiated enrolment. Newspaper articles describing the study and the option of participating will be published in regional newspapers and on the homepage of clinical units in Zurich. Patients initiating contact with the study team via telephone will be screened by the study team, who will carry out ICD-10 diagnosis and study enrolment over the telephone. Additionally, patients need to consult their GP and inform them about study participation. Signed consent forms and baseline questionnaire will be sent via mail (see section “consent and confidentiality”). After the study team receives the signed informed consent and baseline questionnaire, patients are enrolled and subsequently randomised.

Patients allocated to the IC will be called by a member of the study team to arrange an appointment between patient and study therapist ideally within 1 week after randomisation. If a patient is assigned to the CG, the first text message will be sent within a week after randomisation. Four months and 12 months after t0, all patients will receive mail from the study team containing the t1-questionnaire and a stamped envelope to return the completed questionnaire. If a patient fails to return the questionnaire, at first one written reminder for filling out the questionnaire will be sent, and, if still unsuccessful, the patient will be reminded by a phone call. For each completed follow-up questionnaire, patients will receive a CHF 25.-shopping voucher.

## Methods: Assignment of interventions

### Allocation

#### Randomisation and sequence generation

All eligible and consenting patients will be allocated randomly to either IG or CG after completion of baseline measurements. The computer-generated randomisation list with block sizes of 10, which contains the enrolment number and an assigned code for allocation to study condition, was prepared by one of the study team members. A research assistant of the unit not involved in the recruitment procedure prepared sequentially numbered opaque envelopes for each enrolment number separately and placed each enrolment number with the corresponding allocation code of the randomisation list in the according envelope. Study allocation will be concealed by a member of the study team (EH) by opening the envelope that corresponds with the enrolment number and by subsequently contacting the patient via telephone in order to declare the allocation result.

## Methods: Data management and analysis

### Data management

Data coding and entry of the paper-based data will be performed manually by a research assistant trained in standardised coding and being supervised by the study coordinator (EH), who will also check accuracy of data entry by conducting sampling inspections. Data will be stored securely and in a pseudonymised manner. Electronic data will be saved on a secure server with access restriction at the University of Zurich including daily backup and accessible exclusively by the study coordinator and principal investigator. After study completion electronic data will be saved on a separate password protected medium (external hard drive), which will be stored together with print data in a lockable cabinet of the Department of Clinical Psychology and Psychotherapy Research. During study trial as well as after completion of the study Principal Investigator and Study Coordinator exclusively have access to the data. In accordance with the national regulations in Switzerland data will be stored for 10 years after termination of the trial. Since all data will be collected in one study site a data monitoring committee was not established.

### Statistical analysis

Data will be analysed according to the CONSORT statement [39]. The primary analysis will refer to the intention-to-treat (ITT) sample that includes all randomised eligible patients. Multiple imputation of missing data will be performed if data required for the primary analysis are missing. For the primary outcome ‘PHQ-9 change in depressive symptoms from t0 to t2’ an analysis of covariance (ANCOVA) with baseline PHQ-9 as covariate and group as fixed effect will be calculated in order to estimate and test the difference between the IG and the CG. The secondary outcomes will be examined in an exploratory manner with appropriate procedures. Sensitivity analyses will be performed by application of an alternative imputation method (last observation carried forward, LOCF), inclusion of further covariates and restriction to complete cases or to the per protocol population (PP sample) consisting of patients without major violations of the study protocol. The decision whether a protocol violation is judged as major will be blindly met by the study team. Interim analyses are not planned. The significance level will be set at 5% (two-sided). Statistical analyses will be carried out with SPSS, Version 23 (IBM Corp., Armonk, NY, USA).

In order to determine the cost-effectiveness of T-CBT, health care utilisation and productivity loss measured with an adapted version of the CSSRI will be monetarily valued by Swiss unit costs to calculate direct and indirect costs. Quality-adjusted life years (QALYs) will be calculated based on the EQ-5D index [40], and the incremental cost-effectiveness ratio (ICER) will be computed. To adjust for baseline differences and to evaluate the uncertainty of the estimates, net-benefit regressions will be conducted and cost-effectiveness acceptability curves will be derived therefrom.

### Consent and confidentiality

Informed consent will be obtained from all eligible patients before study enrolment: Interested and eligible patients are informed by their GPs or by the study team about the purpose of the study. They are handed out a detailed patient information document and a consent form to take home and read through it carefully. After patients return the signed informed consent, patients will be enrolled into the study. After study enrolment, patients will fill out baseline questionnaire and will subsequently be randomised. The study information document and informed consent form was approved by the Ethics Committee of the Canton of Zurich.

The principal investigator (BW) affirms and upholds the principle of the participant’s right to privacy and that they shall comply with applicable privacy laws. Especially, anonymity of the participants shall be guaranteed when presenting the data at scientific meetings or publishing them in scientific journals. Individual subject medical information obtained as a result of this study is considered confidential and disclosure to third parties is prohibited. Subject confidentiality will be further ensured by utilising subject identification code numbers to correspond to treatment data in the computer files, which means that all data will be collected and stored in a pseudonymised manner. The key to the subject identification code numbers is securely and separately stored from the pseudonymised data.

## Discussion

The presented trial aims at investigating the effectiveness and cost-effectiveness of a telephone-delivered short-term CBT compared to routine care for patients suffering from mild to moderate depressive disorders in primary care. A large impact of the results on clinical practice is to be expected: Given the relevance of depressive disorders and the mis- and undersupply for these disorders in terms of evidence-based treatments, the approach of T-CBT could lead to expanded and more flexible low-threshold psychotherapeutic services. If the study shows that T–CBT is more effective (and cost-effective) than TAU, it could be used to improve the treatment for (primary care) patients suffering of depression. Firstly, a larger proportion of patients in need could be reached with this approach, so the access to psychological care would be enhanced. Secondly, affected patients would receive evidence-based psychological treatments, which are appropriate for their specific conditions. Both factors could result in an improved treatment pathway and outcome. As these effects would be very beneficial for both the individual patient (with less suffering, fewer symptoms and better functional health) as well as for society and the health system (lower direct and indirect costs), an implementation in routine mental health care seems to be indicated in case of positive study results. As the trial will be embedded in routine care, it will also provide information for implementation and roll out.

## Abbreviations

CBT: Cognitive-behavioural therapy
CG: Control group
CSSRI: Client Sociodemographic and Service Receipt Inventory
GP: General practitioner
HrQoL: Health-related quality of life
ICD-10: International Classification of Disease
IG: Intervention group
LOCF: Last observation carried forward
PHQ-9: Patient Health Questionnaire
PP sample: Per protocol sample
QALYs: Quality adjusted life years
TAU: Treatment as usual
T-CBT: Telephone-delivered psychotherapy
